# LncRNA ASAP1-IT1 enhances cancer cell stemness via regulating miR-509-3p/YAP1 axis in NSCLC

**DOI:** 10.1186/s12935-021-02270-7

**Published:** 2021-10-29

**Authors:** Yantao Liu, Yuping Yang, Lingli Zhang, Jiaqiang Lin, Bin Li, Min Yang, Honghui Li, Kangwu Chen, Wei Zhao

**Affiliations:** 1 Department of Pharmacy, West China Second University Hospital, Sichuan University, Chengdu, China; 2 Evidence-Based Pharmacy Center, West China Second University Hospital, Sichuan University, Chengdu, China; 3 Key Laboratory of Birth Defects and Related Diseases of Women and Children, Sichuan University, Ministry of Education, Chengdu, China; 4 Department of Respiratory Medicine, The First Affiliated Hospital of Chengdu Medical College, Chengdu, China; 5 School of Laboratory Medicine Chengdu Medical College, Chengdu, China; 6 Department of Orthopedic Surgery, The First Affiliated Hospital of Soochow University, Suzhou, China; 7 Department of Refractive Surgery, Chengdu Aier Eye Hospital, Chengdu, China; 8 Department of Biomedical Sciences, City University of Hong Kong, Hong Kong, China

**Keywords:** ASAP-IT1, miR-509-3p, YAP1, Cancer stemness, NSCLC

## Abstract

**Background:**

Non-small cell lung cancer (NSCLC) is a major cause of cancer-related death worldwide, and cancer stem cell is responsible for the poor clinical outcome of NSCLC. Previous reports indicated that long noncoding RNAs (lncRNAs) play important roles in maintaining cancer stemness, however, the underlying mechanisms remain unclear. This study investigates the role of ASAP1 Intronic Transcript 1 (ASAP1-IT1) in cancer cell stemness of NSCLC.

**Methods:**

The expression of ASAP1-IT1, microRNA-509-3p (miR-509-3p) and apoptosis-/stemness-related genes was analyzed by qRT-PCR in NSCLC tissues, cancer cells and spheres of cancer stem cells. Knockdown of ASAP1-IT1 or overexpression of miR-509-3p in NSCLC cells by infection or transfection of respective plasmids. Sphere formation and colony formation were used to detect NSCLC stem cell-like properties and tumor growth in vitro. Luciferase reporter assays, RNA immunoprecitation (RIP) and qRT-PCR assays were used to analyze the interaction between lncRNA and miRNA. The expression of expression of regulated genes of ASAP1-IT1/miR-509-3p axis was evaluated by qRT-PCR and Western blot. The NSCLC xenograft mouse model was used to validate the role of ASAP1-IT1 in NSCLC stemness and tumor growth in vivo.

**Results:**

ASAP1-IT1 was up-regulated in NSCLC tissues, cancer cells, and in spheres of A549-derived cancer stem cells. Downregulation of ASAP1-IT1 or overexpression of miR-509-3p significantly decreased cell colony formation and stem cell-like properties of A549-dereived stem cells with decreased expression of stem cell biomarkers SOX2, CD34, and CD133, and suppressing the expression of cell growth-related genes, Cyclin A1, Cyclin B1, and PCNA. Furthermore, knockdown of ASAP1-IT1 or overexpression of miR-509-3p repressed tumor growth in nude mice via reducing expression of tumorigenic genes. ASAP1-IT1 was found to interact with miR-509-3p. Moreover, overexpression of ASAP1-IT1 blocked the inhibition by miR-509-3p on stem cell-like properties and cell growth of A549-dereived stem cells both in vitro and in vivo. Finally, the level of YAP1 was regulated by ASAP1-IT1 and miR-509-3p.

**Conclusions:**

YAP1-involved ASAP1-IT1/miR-509-3p axis promoted NSCLC progression by regulating cancer cell stemness, and targeting this signaling pathway could be is a promising therapeutic strategy to overcome NSCLC stemness.

**Supplementary Information:**

The online version contains supplementary material available at 10.1186/s12935-021-02270-7.

## Background

Non-small cell lung cancer (NSCLC) is the major type of lung cancer which is a leading course of cancer-related death worldwide [1]. Although the treatment of NSCLC has been improved, the 5-years survival rate remains low owing to cancer relapse after surgery or radiotherapy [2, 3]. Therefore, it is critical to better understand the molecular mechanisms of NSCLC progression and find out biomarkers to identify indolent and aggressive tumors, to provide novel therapeutic strategies.

Increasing evidence have demonstrated that the recurrence of NSCLC is mostly induced by cancer stem cells in tumors [4–6]. Cancer stem cells are involved in cancer initiation, proliferation, invasion, and differentiation, resulting in occurrence of aggressive and metastatic cancers [7]. Simultaneously, cancer stem cells can bring about multi-drug resistance in NSCLC [8]. Thus, it is interesting and meaningful to investigate the association of cancer stem cell and NSCLC progression, that would create therapeutic strategies.

Long noncoding RNAs (lncRNAs) are involved in the progression of multiple cancers including NSCLC [9–13]. AFAP1-AS1 (Actin Filament Associated Protein 1 Antisense RNA 1) is a lncRNA which promotes NSCLC cell proliferation by epigenetically suppressing p21 expression [14]. Linc00673 (long intergenic non-protein coding RNA 673) modulates NSCLC cell proliferation, migration, invasion and epithelial mesenchymal transition via sponging miR-150-5p [15]. Linc00473 (long intergenic non-protein coding RNA 473), regulated by cAMP/CREB (adenosine monophosphate/cAMP-response element binding protein), and LKB1 (liver kinase B1) inhibits lung cancer and controls tumor growth [16]. Furthermore, lncRNAs also affect the stemness of lung cancer cells. DGC5 (DiGeorge syndrome critical region gene 5) contributes to cancer cell stemness-like properties by regulating miR-330-5p/CD44 in NSCLC [17]. Linc00662 (long intergenic non-protein coding RNA 662) increases cancer stem cell-like phenotypes in lung cancer [18]. FENDFRR (FOXF1 adjacent non-coding developmental regulatory RNA) inhibits cancer cell stemness by repressing expression of MDR1 (multi-drug resistance gene 1) by interacting with the RNA binding protein HuR in NSCLC development [19]. Linc-ITGB1 (long intergenic non-protein coding RNA integrin subunit beta 1) decreases cancer stemness via down-regulating Snail in NSCLC [20]. HAND2-AS1 (heart and neural crest derivatives expressed 2 antisense RNA 1) elevates cancer cell stemness of lung cancer cells via binding to TGF-beta1 (transforming growth factor beta1) [21]. Interestingly, lncRNA ASAP1-IT1 (ArfGAP with SH3 domain, ankyrin repeat and PH domain 1intronic transcript 1) increases cell proliferation, invasion, and metastasis by regulating PTEN/AKT (phosphatase and tensin homolog/AKT serine/threonine kinase 1) axis in NSCLC [22]. ASAP1-IT1 enhances stemness of cancer cells, and overexpression of ASAP1-IT1 indicates a poor prognosis in patients with bladder cancer [23]. In addition, ASAP1-IT1 promotes development of cholangiocarcinoma through hedgehog signaling pathway [24]. However, the role of ASAP1-IT1 on cancer cell stemness in NSCLC is largely unknown.

In this study, we explored the role of ASAP1-IT1 in NSCLC progression. The binding of ASAP1-IT1with miRNAs was predicted using LncBook database (a curated knowledgebase of human long non-coding RNAs) (https://bigd.big.ac.cn/lncbook/index). We found that miR-509-3p was a potential target of ASAP1-IT1. Previous studies demonstrated that miR-509-3p functions as a tumor suppressor. For instance, miR-509-3p increases platinum drug sensitivity to induce cell apoptosis in ovarian cancer [25, 26]. MiR-509-3p suppresses cell proliferation and migration by up-regulating XIAP (X-linked inhibitor of apoptosis) in gastric cancer [27]. MiR-509-3p represses cell proliferation and invasion by decreasing XIAP in glioma [28]. MiR-509-3p is down-regulated by the oncoprotein HBXIP (late endosomal/lysosomal adaptor, MAPK and MTOR activator 5) via activating NF-κB in hepatoma cells. Additionally, the potential targets of miR-509-3p were predicted using Target Scan tools, and the results showed that YAP1 (Yes associated protein 1) was a downstream target of miR-509-3pin NSCLC cells. YAP1 has been considered as an oncogene in multiple cancers including NSCLC [29–33]. YAP1 positively regulates drug resistance and cancer cell stemness [29, 30]. Also, YAP1is the main effector of the Hippo signaling pathway involved in human cancers [34].

In this study, we investigate the role of ASAP1-IT1, and the association of ASAP1-IT1, miR-509-3p and YAP1 in NSCLC progression and cancer cell stemness.

## Materials and methods

### Clinical specimens

The NSCLC specimens were collected from 83 patients who were treated in the First Affiliated Hospital of Chengdu Medical College (Chengdu, China) from April in 2019 to September in 2020. The collection of clinical samples was approved by the ethics committee of Chengdu Medical College (approved ID: BR20re19), and each participant signed an informed consent form. All the patients were given no treatment before surgery. The collected specimens were stored in a liquid nitrogen tank.

### Cell maintenance and transfection

The A549 and A549/R (cisplatin resistant) cells were obtained from China Infrastructure of Cell Line Resource (Beijing, China), and cultured in DMEM (Dulbecco’s Modified Eagle Medium) media (WISENT, Nanjing, Jiangsu, China) in a CO_2_ incubator at 37 °C. The DMEM media was supplemented with 10% FBS (fetal bovine serum), 100 mg/mL streptomycin and 100 U/mL penicillin (WISENT). The FBS was bought from Thermo Fisher Scientific (Waltham, Massachusetts, USA). All the other cell culture materials were bought from WISENT.

The cell infection was conducted according to the manufacturer’s instruction (Genechem, Shanghai, China). sh-ASAP1-IT1: 5′-GCU GCG ACA AUA GAC AUC GGA GUU U-3′, and sh-NC: 5′-CUC UCG GAA CAU GUC ACA U-3′. The mimic microRNAs and inhibitors were purchased from Ribobio (Guangzhou, China), miR-509-3p-related sequences were listed, Mock, 5′-UCU CCG AAC GUG UCA CGU U-3′; and anti-miR-509-3p, 5′-CCG UGG UUC AUA CUG GUA-3′; miR-509-3p mimic, 5′-UAC CAC AGG GUA GAA CCA CGG-3′. The pmirGLO-ASAP1-IT1-WT (wild type) or -Mut (Mutant), and pmirGLO-Yap1-3′UTR (untranslated region)-WT or -Mut (Mutant), and pcDNA-sh-NC, pcDNA-sh-ASAP1-IT1 were obtained from Thermo Fisher Scientific.

### Hematoxylin–eosin (H&E) staining

The NSCLC specimens were fixed in 4% paraformaldehyde, embedded in paraffin and subjected to sectioning. Then, H&E staining was performed as described previously [10]. All the chemicals and reagents were bought from Servicebio (Wuhan, Hubei, China).

### RNA extraction and qRT-PCR (quantitative real time polymerase chain reaction)

The total RNA was extracted using TRIzol reagent (Life Technologies, Rockville, MD, USA). The reverse transcription kit (Thermo Fisher Scientific) was applied to synthesize cDNA (complementary DNA) from the total RNA. Quantitative RT‐PCRs were used to evaluate the levels of miRNAs, lncRNAs, or mRNAs using the SYBR Premix Ex Taq II (TaKaRa, Dalian, China). The qRT-PCR was carried out under the thermal cycling conditions: 95 °C × 5 min, and 40 cycles of 95 °C × 30 s, 60 °C × 30 s, and 72 °C × 1 min. GAPDH (glyceraldehyde-3-phosphate dehydrogenase) was used as the internal control for RNA and lncRNA, and U6 was used as the internal control of miRNAs. The relative RNA levels were calculated following the 2^−ΔΔCT^ method. The PCR primers were list in Table 1. All experiments were performed in quadruplicate, and each assay was repeated independently for 3 times.

**Table1:** The used PCR primers in this study

Gene name	Forward primer (5′–3′)	Reverse primer (5′–3′)
U6	TGC GGG TGC TCG CTT CGG CAG C	GTG CAG GGT CCG AGG T
miR-509-3p	UAC CAC AGG GUA GAA	CTC TAC AGC TAT ATT GCC AGC CA
GAPDH	CAC CCA CTC CTC CAC CTT TG	CCA CCA CCC TGT TGC TGT AG
Cyclin A1	ATA ACG ACG GGA AGA GCG G	CAG GGT ACA TGA TTG CGG GA
Cyclin B1	CAG GTT GTT GCA GGA GAC CA	CAT GGC AGT GAC ACC AAC CA
PCNA	CGC CCT GGT TCT GGA GGT AA	GGC TGA GAC TTG CGT AAG GG
Bcl-2	TTC TTT GAG TTC GGT GGG GT	GAA ATC AAA CAG AGG CCG CAT
Bax	GGG TTG TCG CCC TTT TCT AC	AGT CGC TTC AGT GAC TCG G
YAP1	TGC TGT CCC AGA TGA ACG TC	GGT TCA TGG CAA AAC GAG GG
ASAP1-IT1	AAA CAT CAT CCC CAG AGT GG	GCC TTG CTC ACC TCT GAA AC
SOX2	CAT GAA GGA GCA CCC GGA TT	ATG TGC GCG TAA CTG TCC AT
CD44	GCC ACC AGA GCT ATT CCC AA	GGT CTT CGC CCA GCC TTT CT
CD133	GCC ATG CTC TCA GCT CTC C	TCC TGA AAA GGA GTT CCC GC

### Western blot

Total protein samples were prepared from the tissues or cells using RIPA (radio immunoprecipitation assay) buffer (Beyotime) for 30 min. The lysates were centrifuged at 12,000×*g* for 15 min to obtain the supernatants, and they were de-natured at 98 °C for 20 min. 50 μg proteins were used for sodium dodecyl sulfate–polyacrylamide gel electrophoresis (SDS-PAGE) (Bio-Rad, Hercules, CA, USA). All the prepared gels were transferred to the 0.22 μm PVDF (polyvinylidene difluoride) membranes (Thermo Fisher Scientific) for 2 h, and the proteins-carried PVDF were blocked with 1% bovine serum albumin. Then, the PVDF membranes were incubated with the primary antibody overnight in a 4 °C refrigerator. The membranes were incubated with the HRP (horseradish peroxidase)-conjugated secondary antibody diluted at 1:50,000 (Bioworld Technology, Nanjing, China; Goat Anti-Rabbit IgG, Cat No. BS10550; Goat Anti-Mouse IgG, Cat No. BS12471) for 1 h. The anti-YAP1 (Cat No. MA5-32117), anti-β-Actin (Cat No. PA1-183-HRP), anti-Bax (Cat No. MA5-14003), anti-Bcl-2 (Cat No. MA5-11757), and Cleaved-caspase-3 (Cat No. MA5-32134) were bought from Thermo Fisher Scientific.

### Sphere formation and colony formation of cancer stem cells

Cancer stem cells were sorted from A549 cells or transfected-A549 cells by sphero-cyst medium [10], and the sorted cancer stem cells from A549 were named as A549-derived stem cells. The 3 × 10^4^ cells were seeded in 6-well ultra-low cluster plates (Thermo Fisher Scientific). They were maintained in DMEM/F12 serum free medium (Thermo Fisher Scientific) containing epidermal growth factor (20 ng/mL), beta-fibroblast growth factor (20 ng/mL), insulin (4 μg/mL), and B27 (2%). All reagents were from Sigma. Finally, the number of spheres was counted under an inverted microscope (Leica, Oskar-Barnack-Straße, Germay) post 10 days incubation. The biomarkers of cancer stem cells SOX2, CD34, and CD133 were used to identify the cancer stem cells using qRT-PCR and Western blot.

The A549 cancer stem cells were isolated using magnetic bead kit, and 3 × 10^4^ stem cells were seeded in six-well plates with ultra-low adhesion. They were cultured in sphere formation media for a week, and post another 20 days, the number of spheres was calculated under a microscope.

### Cell apoptosis and cell cycle analysis

To determine cell apoptosis of A549-dereived stem cells and other cells, cells were collected at 80 g × 4 °C × 5 min for annexin-V/FITC (fluoresceine isothiocyanate)/PI (propidium iodide) staining after 48 h infection with the indicated plasmids or miRNAs. Briefly, cells were incubated with annexin-V and PI for 10 min and 5 min, respectively, in a dark room using the annexin V-FITC/PI staining kit (Beyotime, Beijing, China). Finally, the cell cycle was evaluated using the Beckman Coulter Navios EX flow cytometry (Beckman, Shanghai, China).

For analyzing cell cycle, the cells were cultured in 12-well plates (Thermo Fisher Scientific). After transfection with the indicated miRNAs or plasmids for 48 h, they were subjected to suspension and fixation in 75% ethanol at 4 °C for 12 h. Then, they were washed with PBS (phosphate‐buffered saline) for 3 times. Afterwards, they were resuspended in 500 μL staining buffer containing propidium iodide (5 mg/mL)/RNase (10 mg/mL) at 37 °C for 30 min in a dark room. Finally, cell apoptosis rate was evaluated using the flow cytometry. Each treatment group consisted four wells, and each assay was repeated for 3 times independently.

### Cell counting kit‐8 (CCK-8) assay

The A549-dereived stem cells or A549 cells were plated into a 96-well plate (Thermo Fisher Scientific) at the density of 4 × 10^3^ cells per well for determination of cell viability. CCK‐8 reagent (Sangon, Shanghai, China) were added into medium after the cells were cultured 36 h. After incubation for 1 h at 37 °C, the formation of water‐soluble formazan was examined in a microplate reader (Bio-Rad) with the light length of 450 nm. All experiments were performed in quadruplicate, and each assay was repeated independently for 3 times.

### Dual luciferase reporter assay

To determine the effects of miR-509-3p on luciferase activity of pmirGLO-ASAP1-IT1, 2 × 10^4^ A549 cells per well were seeded in 24-well plates. They were co-transfected with pmirGLO-ASAP1-IT1-WT (wild type), pmirGLO-ASAP1-IT1-Mut (Mutant), as well as Mock (negative control), miR-509-3p mimic, and anti-miR-509-3p. To examine the effects of miR-509-3p on luciferase activity of pmirGLO-Yap1-3′UTR, the cells were co-transfected with pmirGLO-Yap1-3′ UTR-WT, pmirGLO-Yap1-3′ UTR-Mut (Mutant), as well as Mock, miR-509-3p mimic, and anti-miR-509-3p. After transfection for 48 h, they were collected and lysed. The supernatants were used for measurement of luciferase activity using the Dual-Luciferase Assay Kit on GloMax 20/20 Luminometer following the manufacturer’s instructions (Promega, Madison, USA). All experiments were performed in quadruplicate, and each assay was repeated independently for 3 times.

### RNA immunoprecipitation (RIP) assay

RIP assay was conducted to examine the interaction between ASAP1-IT1 and miR-509-3p using the RIP RNA-binding protein immunoprecipitation kit Magna (Millipore, Massachusetts, USA). Briefly, the cell lysate was incubated with anti-Ago2 (argonaute-2), and anti-IgG was used as a negative control. The antibodies were purchased from Bioworld Technology (Nanjing, China). At last, the collected immunoprecipitated RNA samples were used to determine ASAP1-IT1 and miR-1301-3p content by qRT-PCR analysis. All experiments were performed in quadruplicate, and each assay was repeated independently for 3 times.

### Mouse tumorigenesis assay

The A549-dereived stem cells were infected with the mentioned shRNAs or plasmids as indicated. Post 48 h infection, the A549-dereived stem cells were collected at 80 g × 4 °C × 5 min. Afterwards, 4 × 10^6^ cells were inoculated into 5-week-old nude mice. The BALB/c nude mice were bought from the Model Animal Research Center of Nanjing University (Nanjing, China), and divided randomly with 3 mice in each group. The animal experiments were approved by the research ethics committee of Chengdu Medical College (approved animal protocol No. CMC20LM22). The tumor volume was calculated every 3 days using the formula, tumor volume = (length × width^2^)/2. All the tumor-carried nude mice were sacrificed on the 24th day post inoculation.

### Statistical analysis

Statistical analysis was conducted using SPSS software package (version 20.0, SPSS Inc., NY, USA) and GraphPad Prism 6 (GraphPad Software, CA, USA). A p value < 0.05 was considered statistically significant. The data were obtained from three independent experiments, and all data were expressed as mean ± standard deviation (S.D.). The statistical significance was examined using Two-tailed Student’s t-test for two-group comparisons and one-way analysis of variance (ANOVA) test with post-hoc analysis for multi-group comparisons.

## Results

### ASAP1-IT1 is overexpressed in NSCLC tissues and knockdown of ASAP1-IT1 inhibited stemness of NSCLC cells

**Fig. 1:**
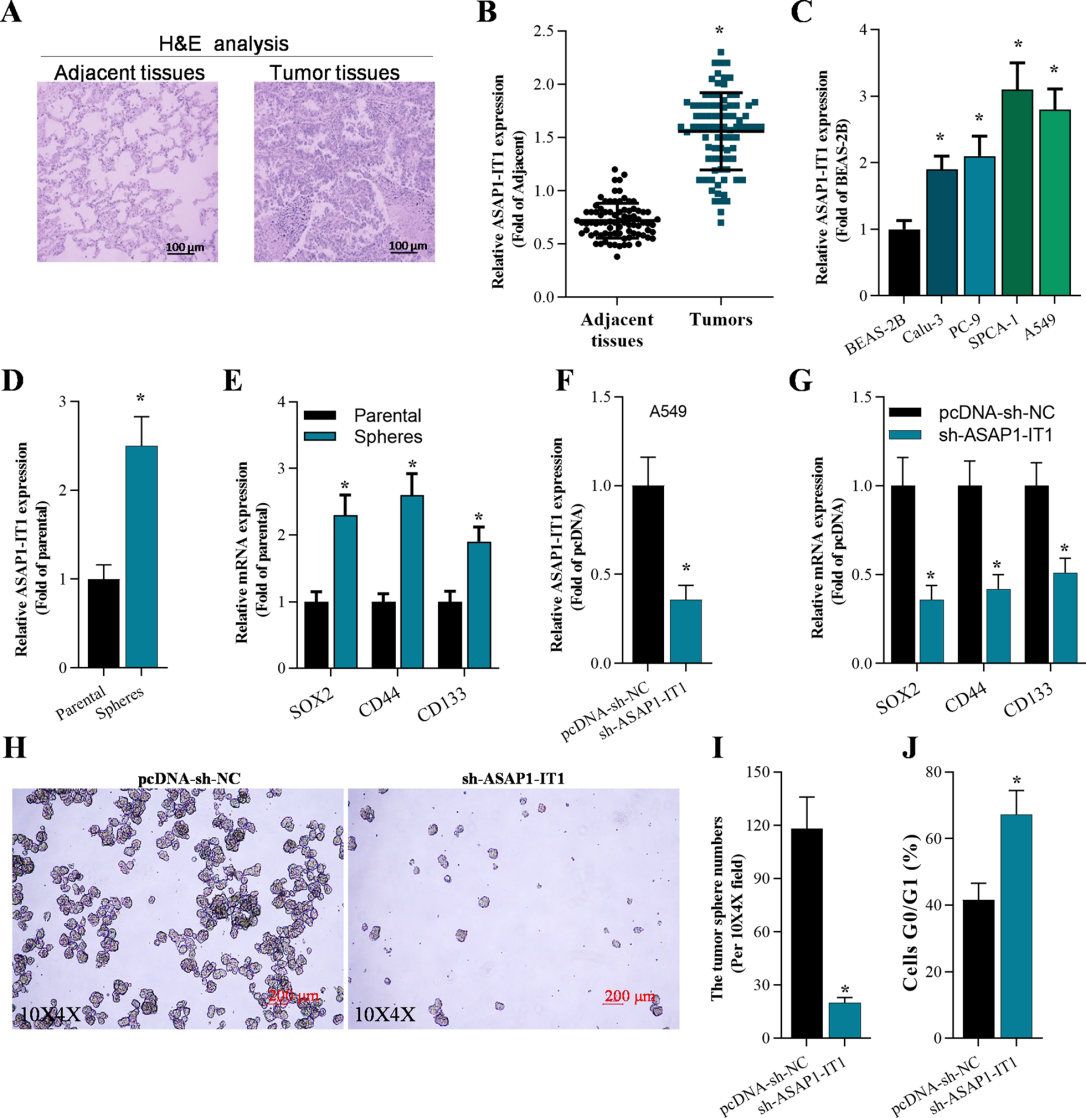
ASAP1-IT1was elevated in NSCLC tissues and knockdown of ASAP1-IT1inhibited the cancer cell stemness of NSCLC. **A** H&E staining of NSCLC tissues and adjacent tissues. **B** qRT-PCR analysis of ASAP1-IT1 expression in NSCLC tissues and adjacent tissues, *p < 0.01 compared with adjacent tissues (n = 83); **C** qRT-PCR analysis of ASAP1-IT1 expression in NSCLC cells and normal cells, *p < 0.01 compared with BEAS-2B cells; **D** qRT-PCR analysis of ASAP1-IT1 expression in spheres of A549, *p < 0.01 compared with parental group. **E** qRT-PCR on the expression of stemness-associated gens (SOX2, CD44, and CD133) in stemness‐enriched cell spheres and parental cells, *p < 0.01 compared with parental group. **F** qRT-PCR analysis of ASAP1-IT1 levels after 48 h infection with pcDNA-sh-NC or pcDNA-sh-ASAP1-IT1 in A549-derived stem cells, *p < 0.01 compared with pcDNA-sh-NC. **G** qRT-PCR analysis on stemness-associated gens (SOX2, CD44, and CD133) after 48 h infection with pcDNA-sh-NC or pcDNA-sh-ASAP1-IT1 in A549-derived stem cells, *p < 0.01 compared with pcDNA-sh-NC. **H**, **I** Number of tumor spheres in ASAP1-IT1 knockdown A549-derived stem cells, *p < 0.01 compared with pcDNA-sh-NC. **J** Flow cytometry analysis of cell cycle in A549 stem cells post 48 h infection, *p < 0.01 compared with pcDNA-sh-NC

To evaluate the role of ASAP1-IT1 in NSCLC, we determined the ASAP1-IT1 expression in human NSCLC specimens and adjacent tissues using qRT-PCR. The H&E staining confirmed the dysregulated cancerous growth in NSCLC tissues (Fig. 1A). The qRT-PCR results demonstrated that ASAP1-IT1 was significantly overexpressed in NSCLC tissues compared with that in adjacent tissues (Fig. 1B), High expression of ASAP1-IT1 (> mean value of all tumors) was positively correlated with tumor differentiation, TNM stage, and lymph node metastasis (Table 2). Meanwhile, ASAP1-IT1 was also up-regulated in NSCLC cells (A549, Calu-3, PC-9, and SPCA-1) (Fig. 1C). To investigate the role of ASAP1-IT1 in inducing stemness in NSCLC, we determined the ASAP1-IT1 expression between parental A549 cells and stemness-enriched A549 spheres. The qRT-PCR data showed that ASAP1-IT1 was markedly elevated in spheres of A549-dereived stem cells compared with the parental group (Fig. 1D). Meanwhile, stemness-associated genes including SOX2, CD44, CD133 were increased in A549 spheres compared with that in parental cells (Fig. 1E). To explore the function of ASAP1-IT1 in stemness formation, the ASAP1-IT1 expression was knocked down in A549-dereived stem cells (Fig. 1F). The qRT-PCR analysis showed that knockdown of ASAP1-IT1 significantly down-regulated expression of stemness-associated genes SOX2, CD44, and CD133 in A549-dereived stem cells (Fig. 1G). Furthermore, suppression of ASAp1-IT1 significantly reduced sphere formation in comparison to the pcDNA-sh-NC group (Fig. 1H, I). Additionally, downregulation of ASAP1-IT1 arrested A549-dereived stem cells in G0/G1 phase (Fig. 1J).

**Table 2:** Association between lncRNA ASAP1-IT1 expression to clinical characteristics in the NSCLC tissues

Factor	ASAP1-IT1 level (low)	ASAP1-IT1 level (high)	*P*-value
Sex			0.327
Male	22	25	
Female	17	19	
Age			0.431
≤ 60	23	24	
> 60	18	18	
Smoking status			
Yes	20	24	0.297
No	17	22	
Histology			0.506
SSC	21	23	
AC	20	19	
Others	0	0	
Tissue differentiation			0.012
Middle and high	6	37	
Low	8	32	
TNM stage			0.015
I/II	12	34	
III/IV	7	30	
Lymph node metastasis			0.013
Present	14	28	
Absent	11	30	

*ASAP1-IT1* ArfGAP with SH3 domain, ankyrin repeat and PH domain 1 intronic transcript 1, *NSCLC* non-small cell lung cancer, *SCC* lung squamous cell carcinoma, *AC* lung adenocarcinoma

A *P* value < 0.05 was statistically significant

### Knockdown of ASAP1-IT1 suppressed cell growth and reduced cisplatin resistance in cancer stem cells, and promoted apoptosis

**Fig. 2:**
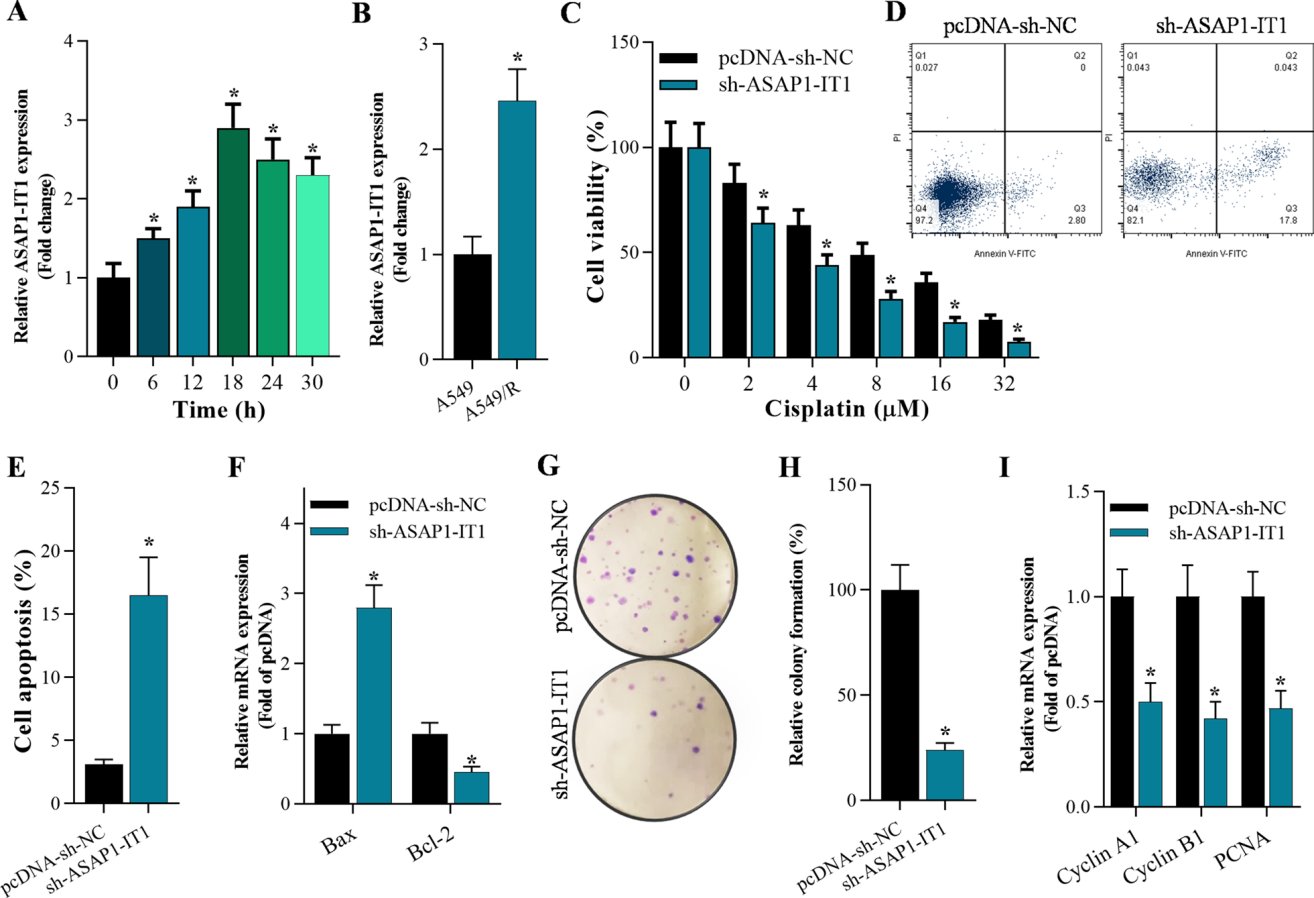
Knockdown of ASAP1-IT1suppressed cisplatin resistance and cell growth of cancer stem cells and interfering ASAP1-IT1promoted apoptosis. qRT-PCR analysis of ASAP1-IT1 expression in **A** A549 cells post incubation with 4 μM cisplatin at different times (0 h, 6 h, 12 h, 18 h, 24 h, 30 h), *p < 0.01 compared with 0 h group and **B** A549 and cisplatin-resistant A549/R cells, *p < 0.01 compared with A549. **C** CCK-8 assay on the cell viability of ASAP1-IT1 knockdown A549/R cells incubated with cisplatin at various concentration (0 μM, 4 μM, 8 μM, 16 μM, 32 μM), *p < 0.01 compared with pcDNA-sh-NC. **D**, **E** Annexin V-FITC/PI staining of A549-dereived stem cells 48 h post infection, *p < 0.01 compared with pcDNA-sh-NC. **F** qRT-PCR analysis of expression of apoptosis-associated genes (Bax and Bcl-2) 48 h after infection in A549-dereived stem cells, *p < 0.01 compared with pcDNA-sh-NC. **G**, **H** Colony formation assay on cell growth of A549-dereived stem cells after 15 days of culture, *p < 0.01 compared with pcDNA-sh-NC. **I** qRT-PCR analysis on the expression of proliferation-associated genes (Cyclin A1, Cyclin B1, and PCNA) after 48 h infection in A549-dereived stem cells, *p < 0.01 compared with pcDNA-sh-NC

Cancer stem cells (CSCs) are closely associated with chemo-resistance, which often reduces therapeutic effects of anti-cancer drugs, such as cisplatin. To examine whether ASAP1-IT1 serves as a dominant factor of cisplatin resistance in NSCLC cells, A549 cells with ASAP1-IT1-knockdown were incubated with 4 μM cisplatin. The qRT-PCR data showed that ASAP1-IT1 was increased after incubation with cisplatin in a time-dependent manner (Fig. 2A). Also, ASAP1-IT1 was up-regulated in A549/R cells compared with that in A549 cells (Fig. 2B). ASAP1-IT1-knockdown A549 cells were then incubated with cisplatin at different concentration, and the CCK-8 assays demonstrated that increasing concentration of cisplatin significantly decreased cell viability of A549 cells with ASAP1-IT1-knockdown (Fig. 2C). Moreover, annexin V‐FITC/PI assays and qRT-PCR analysis confirmed that ASAP1-IT1 knockdown obviously increased apoptotic cells of A549 by elevating expression of Bax and caspase-3, and inhibiting Bcl-2 expression (Fig. 2D–F). Knockdown of ASAP1-IT1 also repressed colony formation of A549-dereived stem cells (Fig. 2G, H), and silencing ASAP1-IT1 expression significantly decreased expression of cell growth-associated genes including Cyclin A1, Cyclin B1, and PCNA (Fig. 2I).

### ASAP1-IT1 reciprocally interacted with miR-509-3p

**Fig. 3:**
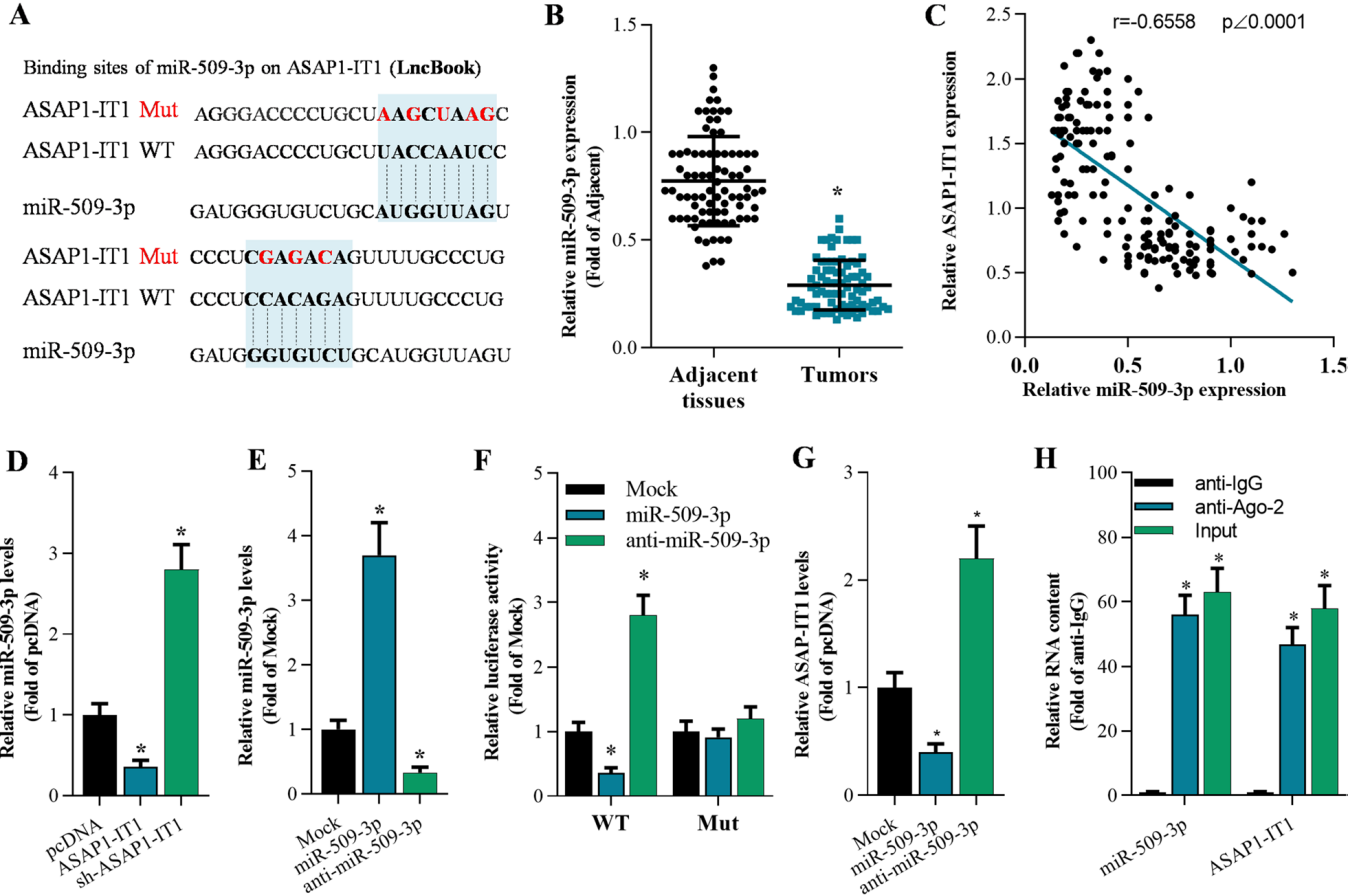
ASAP1-IT1 interacted with miR-509-3p. **A** The potential binding sites of miR-509-3p on ASAP1-IT1 predicted by LncBook. **B** qRT-PCR analysis of miR-509-3p expression in NSCLC tissues and adjacent tissues (n = 83), *p < 0.01 compared with adjacent tissues. **C** The correlation of ASAP1-IT1 and miR-509-3p in NSCLC tumors (n = 83). **D** qRT-PCR analysis of miR-509-3p in A549 cells 48 h post infection with pcDNA, pcDNA-ASAP1-IT1 (ASAP1-IT1), and pcDNA-sh-ASAP1-IT1 (sh-ASAP1-IT1), ^&^*p* < 0.01 compared with pcDNA. **E** qRT-PCR analysis of miR-509-3p expression in A549 cells 48 h after transfection with mimic miRNAs, *p < 0.01 compared with Mock. **F** Luciferase reporter assay onmiR-509-3p on luciferase activity of pmirGLO-ASAP1-IT1 WT (wild type) and pmirGLO-ASAP1-IT1 Mut (mutant) 48 h post transfection with mimic miRNAs, *p < 0.01 compared with Mock. **G** qRT-PCR analysis of ASAP1-IT1 expression in A549 cells 48 h after infection, *p < 0.01 compared with Mock. **H** qRT-PCR analysis of RNA content after RIP assay in A549 cells, *p < 0.01 compared with anti-IgG

To find out the molecular mechanisms of ASAP1-IT1 in regulating NSCLC progression, we predicted its potential interaction with miRNAs using LncBook database (a curated database of human long non-coding RNAs) (https://bigd.big.ac.cn/lncbook/index). It was found that miR-509-3p might be a target miRNA of ASAP1-IT1 (Fig. 3A). In the meantime, miR-509-3p was found to be downregulated in NSCLC tissues, and its expression was negatively correlated with ASAP1-IT1 (Fig. 3B, C). In addition, ASAP1-IT1 significantly suppressed miR-509-3p expression in A549 cells (Fig. 3D). Furthermore, miR-509-3p inhibited luciferase activity of pmirGLO-ASAP1-IT1-WT, and miR-509-3p failed to affect the luciferase activity of pmirGLO-ASAP1-IT1-Mut, which contained the mutated binding sites of miR-509-3p on ASAP1-IT1 (Fig. 3E, F). Moreover, overexpression of miR-509-3 repressed ASAP1-IT1 level while inhibition of miR-509-3p elevated ASAP1-IT1 expression (Fig. 3G).To confirm the direct interaction between miR-509-3p and ASAP1-IT1, RIP assay was conducted. The results supported that ASAP1-IT1 could interact with miR-509-3p in A549 cells (Fig. 3H).

### Overexpression of miR-509-3p inhibited cancer cell stemness, cisplatin resistance and cell growth of A459 stem cells, and promoted apoptosis

**Fig. 4:**
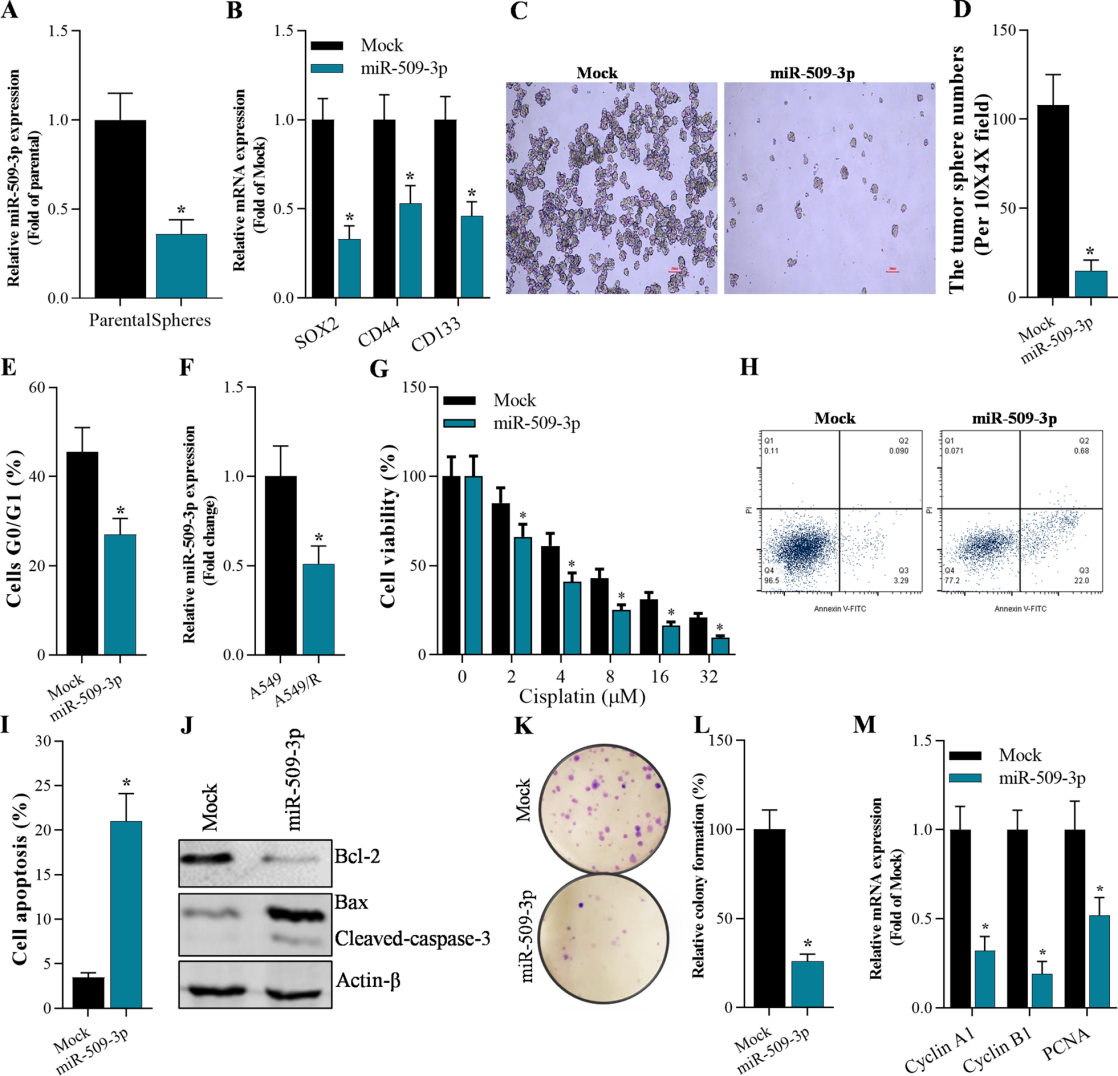
Overexpression of miR-509-3p inhibited stemness, cisplatin resistance and cell growth, and promoted apoptosis of A549-dereived stem cells. **A** qRT-PCR analysis on miR-509-3pin spheres of A549-derived stem cells, *p < 0.01 compared with parental group. **B** qRT-PCR on the expression of stemness-associated gens (SOX2, CD44, and CD133) in A549cell spheres 48 h after transfection with mimic miRNAs, *p < 0.01 compared with Mock. **C**, **D** Number of tumor spheres in miR-509-3p overexpressing A459 stem cells, *p < 0.01 compared with Mock. **E** Flow cytometry analysis of cell cycle in A549-dereived stem cells 48 h after overexpression of miR-509-3p, *p < 0.01 compared with Mock. **F** qRT-PCR analysis of miR-509-3pin A549 and cisplatin-resistant A549/R cells. *p < 0.01 compared with A549. **G** CCK-8 assay on the cell viability of miR-509-3p-overexpressing A549-dereived stem cells treated with cisplatin at various concentration (0 μM, 4 μM, 8 μM, 16 μM, 32 μM), *p < 0.01 compared with Mock. **H**, **I** Annexin V-FITC/PI staining of A549-dereived stem cells 48 h after miR-509-3p overexpression, *p < 0.01 compared with Mock. **J** Western blotting analysis on expression of apoptosis-associated genes (Bax, Bcl-2 and cleaved-caspase-3) 48 h after transfection with miR-509-3p or Mock, *p < 0.01 compared with Mock. **K**, **L** Colony formation assay on cell growth of A549-dereived stem cells after 15 days culture, *p < 0.01 compared with Mock. **M** qRT-PCR analysis on proliferation-associated genes (Cyclin A1, Cyclin B1, and PCNA) 48 h after overexpression of miR-509-3p in A549-dereived stem cells, *p < 0.01 compared with Mock

The roles of miR-509-3p in cancer cell stemness, cisplatin resistance, cell growth, and cell apoptosis were investigated both in vitro and in vivo. Quantitative RT-PCR analysis demonstrated that miR-509-3p was reduced in the spheres of A549 cells compared with the parental cells (Fig. 4A). Meanwhile, overexpression of miR-509-3p significantly inhibited stemness-associated genes SOX2, CD44, and CD133 in A549-dereived stem cells compared with that in the Mock group (Fig. 4B). Overexpression of miR-509-3p suppressed sphere formation in comparison to Mock-transfected A549-dereived stem cells (Fig. 4C, D). MiR-509-3p markedly arrested A549-dereived stem cells in G0/G1 phase (Fig. 4E). MiR-509-3p was decreased in A549/R cells compared with A549 cells (Fig. 4F), indicating that miR-509-3p functions as an essential regulator of chemo-resistance in NSCLC cells. Furthermore, miR-509-3p-overexpressed A549-dereived stem cells were incubated with cisplatin at different concentration, and CCK-8 assays showed that increasing concentration of cisplatin significantly decreased cell viability of miR-509-3p-overexpressed A549-dereived stem cells (Fig. 4G). Annexin V‐FITC/PI assays and qRT-PCR analysis showed that miR-509-3p-overexpression markedly elevated apoptosis of A549-dereived stem cells by promoting expression of Bax and cleaved-caspase-3, and down-regulating Bcl-2 expression (Fig. 4H–J). Furthermore, miR-509-3p-overexpression significantly decreased colony formation of A549-dereived stem cells by inhibiting expression of cell growth-associated genes Cyclin A1, Cyclin B1, and PCNA (Fig. 4K–M).

### Overexpression of ASAP1-IT1 blocked the effect of miR-509-3p on cancer stem cells both in vitro and in vivo

**Fig. 5:**
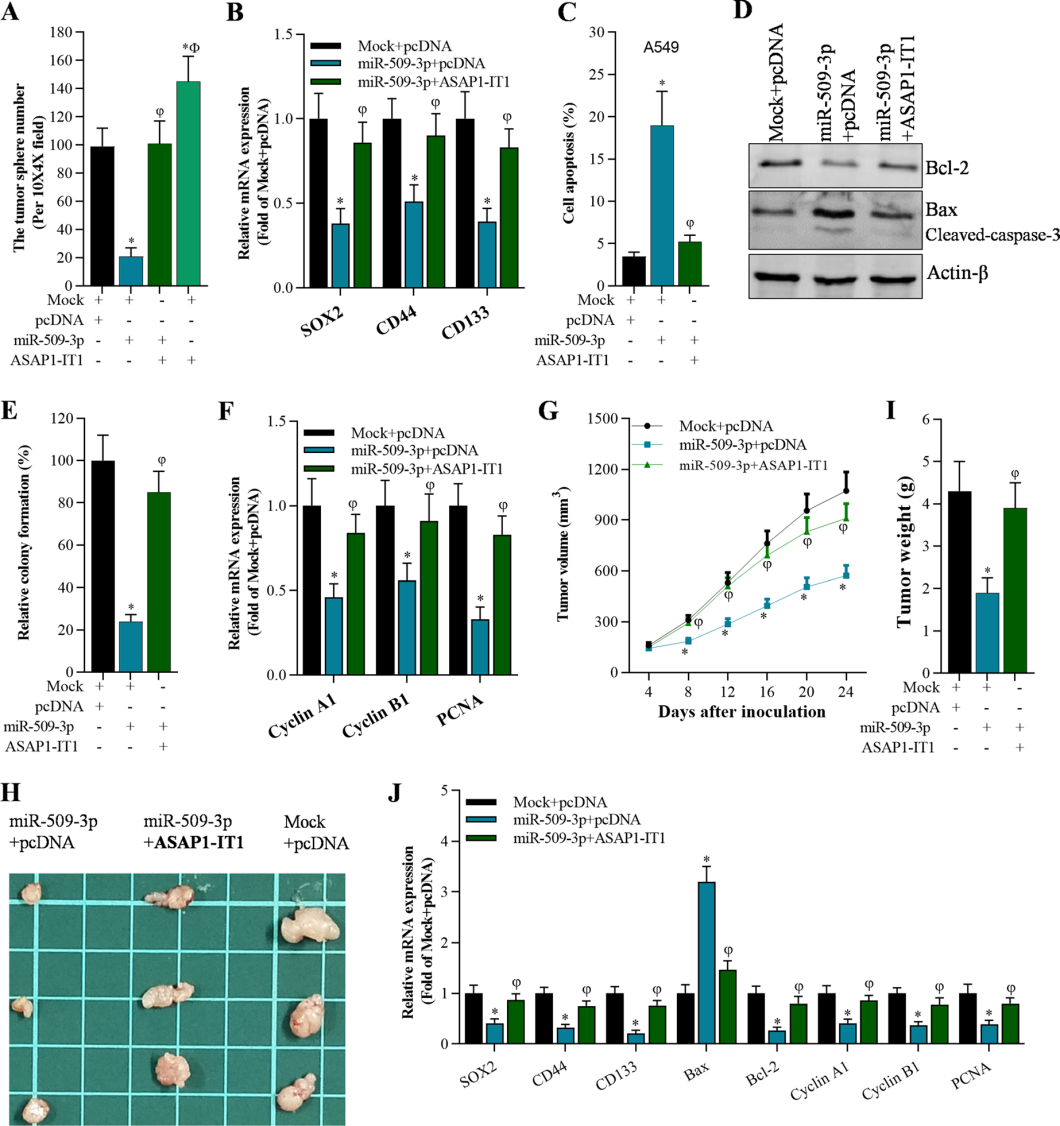
Overexpression of ASAP1-IT1 blocked miR-509-3p-mediated cell functions in cancer stem cells both in vitro and in vivo*.* **A** Number of A549 cell tumor spheres after expression of Mock, pcDNA, miR-509-3p, and ASAP1-IT1, *p < 0.01 compared with Mock + pcDNA, φ*p* < 0.01 compared with miR-509-3p + pcDNA, Ф*p* < 0.01 compared with miR-509-3p + ASAP1-IT1. **B** qRT-PCR analysis on stemness-related genes (SOX2, CD44, and CD133) in A549 stem cells, *p < 0.01 compared with Mock + pcDNA, φ*p* < 0.01compared with miR-509-3p + pcDNA. **C** Annexin V-FITC/PI staining of A549 stem cells with Mock, pcDNA, miR-509-3p, and ASAP1-IT1, *p < 0.01 compared with Mock + pcDNA, φ*p* < 0.01 compared with miR-509-3p + pcDNA. **D** Western blotting analysis on apoptosis-associated genes (Bax, Bcl-2, and cleaved-caspase-3) in A549 stem cells, *p < 0.01 compared with Mock + pcDNA, φ*p* < 0.01 compared with miR-509-3p + pcDNA. **E** Colony formation assay on cell growth after 15 days culture, *p < 0.01 compared with Mock + pcDNA, φ*p* < 0.01 compared with miR-509-3p + pcDNA. **F** qRT-PCR analysis of growth-associated genes (Cyclin A1, Cyclin B1, and PCNA) in A549 stem cells, *p < 0.01 compared with Mock + pcDNA, φ*p* < 0.01 compared with miR-509-3p + pcDNA. **G** Tumor volume in nude mice at the indicated time after inoculation, *p < 0.01 compared with Mock + pcDNA, φ*p* < 0.01 compared with miR-509-3p + pcDNA. **H** Photo of harvested tumors at the 24th day after inoculation. **I** Tumor weight at the 24th day after inoculation, *p < 0.01 compared with Mock + pcDNA, φ*p* < 0.01 compared with miR-509-3p + pcDNA. **J** qRT-PCR analysis on stemness-associated genes (SOX2, CD44, and CD133), apoptosis-associated genes (Bax and Bcl-2), and growth-associated genes (Cyclin A1, Cyclin B1, and PCNA) in tumors, *p < 0.01 compared with Mock + pcDNA, φ*p* < 0.01 compared with miR-509-3p + pcDNA

Based on the above results, overexpression of miR-509-3p reduced the stemness, cisplatin resistance and growth, and promoted apoptosis of A549-dereived stem cells both in vitro and in vivo. Analysis of sphere formation indicated that overexpression of ASAP1-IT1 significantly overturned the effect of miR-509-3p in sphere formation and restored expression of stemness-associated genes including SOX2, CD44, and CD133 (Fig. 5A, B). ASAP1-IT1 overexpression reversed miR-509-3p-induced apoptosis by restoring miR-509-3p-controlled expression of apoptotic genes (Fig. 5C, D). Similarly, ASAP1-IT1 overexpression abolished the suppressive effect of miR-509-3p on cell growth by recovering miR-509-3p-mediated expression of cell growth-related genes including Cyclin A1, Cyclin B1, and PCNA (Fig. 5E, F).

Moreover, it was found that overexpression of miR-509-3p significantly decreased tumor growth whereas overexpression of ASAP1-IT1 could block the miR-509-3p-mediated inhibitory activity on tumor growth in vivo xenografts (Fig. 5G–I). Finally, ASAP1-IT1 also reversed miR-509-3p-controlled expression of genes involved in stemness, cell growth, and cell apoptosis in tumors (Fig. 5J).

### YAP1 is regulated by miR-509-3p and ASAP1-IT1

**Fig. 6:**
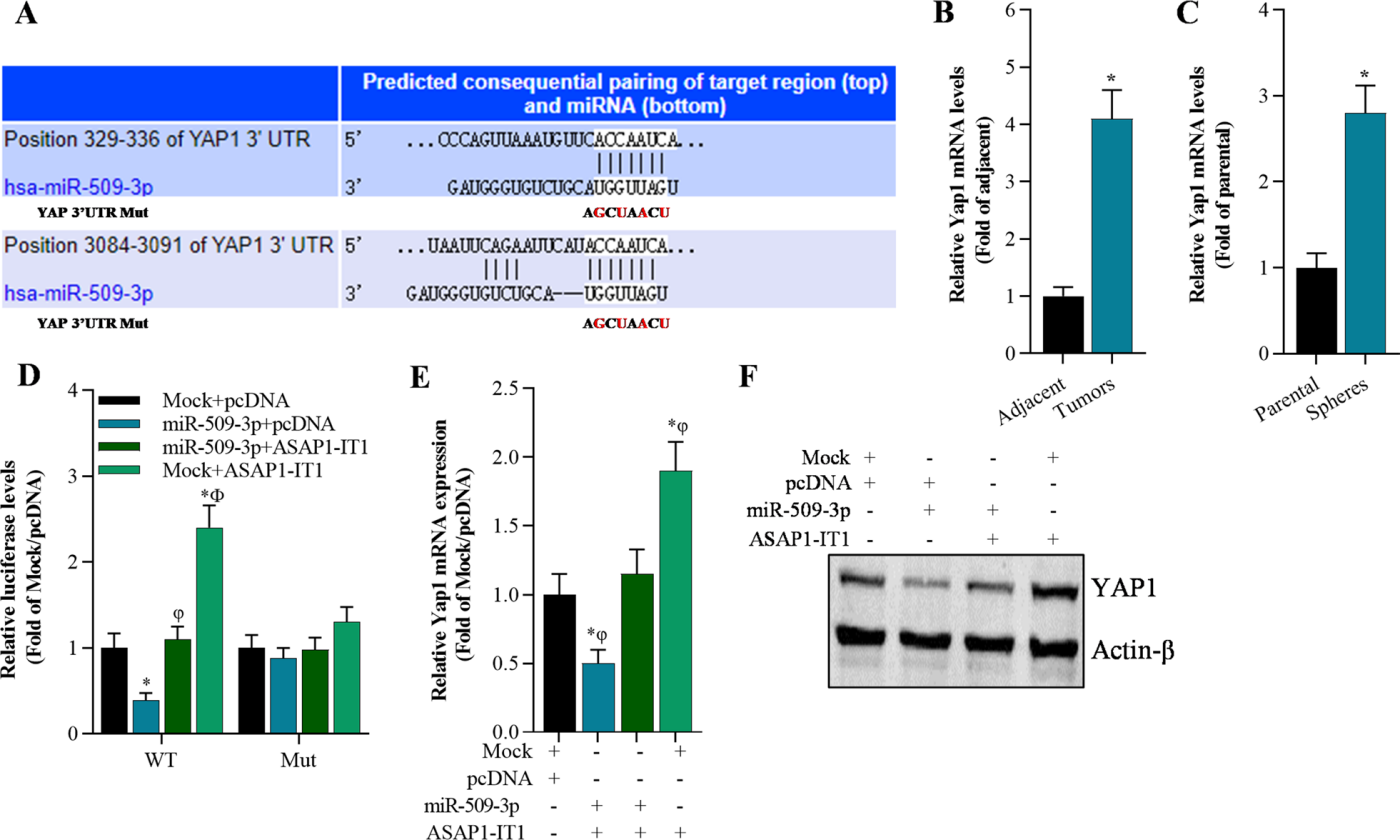
YAP1 is regulated by miR-509-3p and ASAP1-IT1. **A** The potential target of miR-509-3p is predicted by TargetScan. **B** qRT-PCR analysis of YAP1 expression in NSCLC tissues and adjacent tissues, *p < 0.01 compared with adjacent tissues. **C** qRT-PCR analysis of YAP1 in A549 spheres, *p < 0.01 compared with parental group. **D** Luciferase activity of pmirGLO-YAP1-3′UTR-WT (wild type) or pmirGLO-YAP1-3′UTR-Mut (mutant) was modulated by miR-509-3p and ASAP1-IT1 in A549 cells, *p < 0.01 compared with Mock + pcDNA, φ*p* < 0.01 compared with miR-509-3p + pcDNA, Ф*p* < 0.01 compared with miR-509-3p + ASAP1-IT1. **E** YAP1 mRNA levels in A549 cells analyzed byqRT-PCR, *p < 0.01 compared with Mock + pcDNA, φ*p* < 0.01 compared with miR-509-3p + pcDNA, Ф*p* < 0.01 compared with miR-509-3p + ASAP1-IT1. **F** Western blot analysis on YAP1 protein in A549 cells

To further explore the potential molecular mechanism of ASAP1-IT1 and miR-509-3p interaction in NSCLC progression, we explored the downstream molecule of miR-509-3p using online bioinformatics analysis. YAP1 was identified as a target gene of miR-509-3p (Fig. 6A). YAP1 was elevated in NSCLC tissues compared with that in adjacent tissues by qRT-PCR analysis (Fig. 6B). Meanwhile, qRT-PCR analysis also revealed that YAP1 was up-regulated in spheres compared with adherent cancer cells (Fig. 6C). Luciferase reporter assay demonstrated that miR-509-3p suppressed luciferase activity of pmirGLO-YAP1-3′UTR-WT, and it failed to affect the luciferase activity of pmirGLO-YAP1-3′UTR-Mut which contained the mutated binding sites of miR-509-3p on YAP1 3′UTR (Fig. 6D). In addition, overexpression of miR-509-3p significantly reduced YAP1expression at the mRNA and protein levels (Fig. 6E, F). Moreover, overexpression of ASAP1-IT1 enhancedYAP1 expression (Fig. 6D–F), and significantly blocked miR-509-3p-controlled YAP1 expression evidenced by luciferase reporter assay, qRT-PCR, and Western blot (Fig. 6D–F).

### Overexpression of YAP1 reversed miR-509-3p function on cancer cell stemness, cell growth, and apoptosis in A549-derived stem cells

**Fig. 7:**
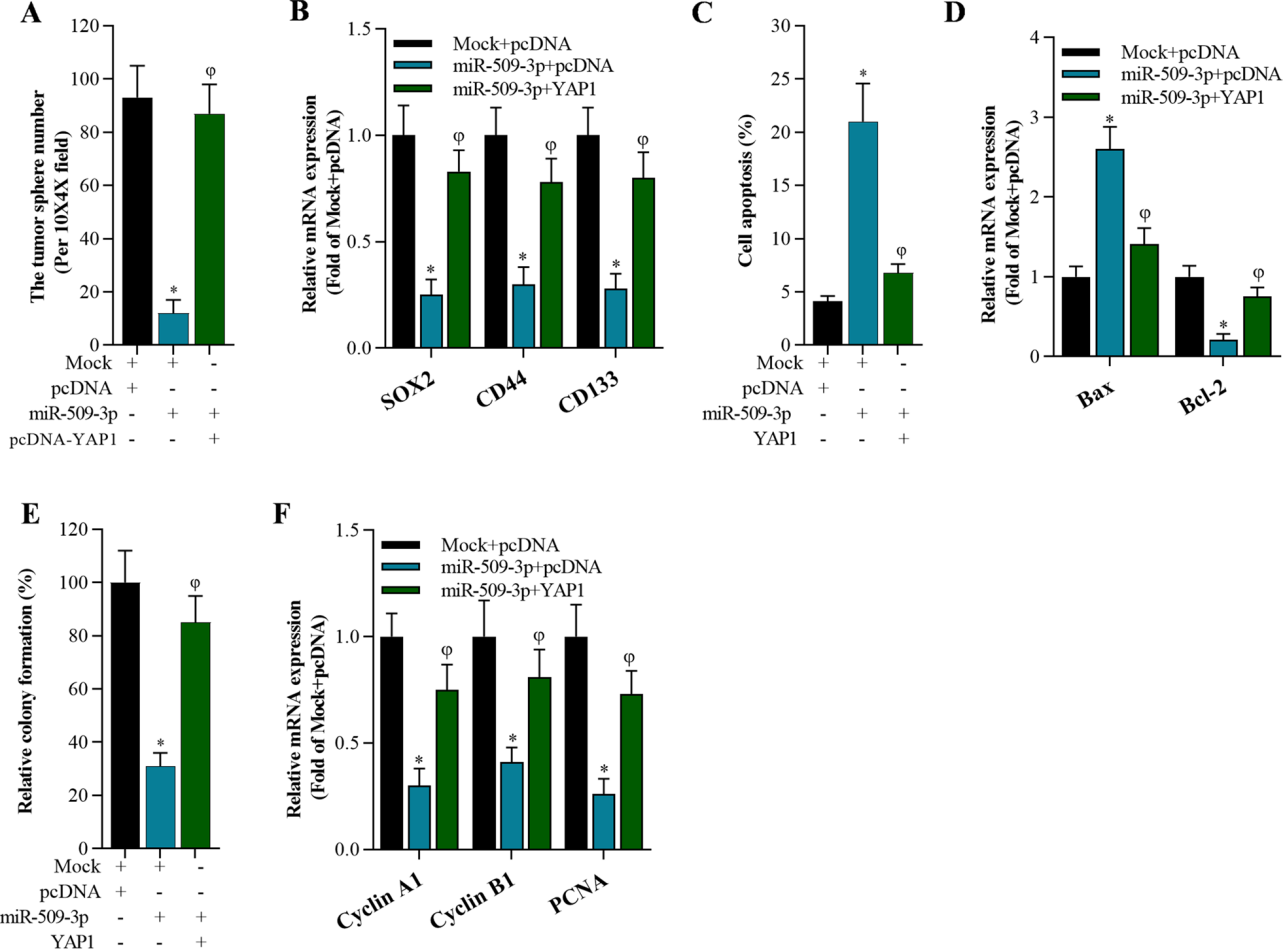
Overexpression of YAP1 reversed miR-509-3p-mediated cancer cell stemness, cell growth, and apoptosis of A549-derived stem cells. **A** Number of tumorA549 spheres after transfection with Mock, pcDNA, miR-509-3p, and YAP1, *p < 0.01 compared with Mock + pcDNA, φ*p* < 0.01 compared with miR-509-3p + pcDNA. **B** qRT-PCR analysis on stemness-related genes (SOX2, CD44, and CD133)48 h after transfection in A549-derived stem cells, *p < 0.01 compared with Mock + pcDNA, φ*p* < 0.01 compared with miR-509-3p + pcDNA. **C** Annexin V-FITC/PI staining of A549-derived stem cells after transfection with Mock, pcDNA, miR-509-3p, and YAP1, *p < 0.01 compared with Mock + pcDNA, φ*p* < 0.01 compared with miR-509-3p + pcDNA. **D** qRT-PCR analysis on apoptosis-associated genes (Bax, Bcl-2, and caspase-3) in A549-derived stem cells, *p < 0.01 compared with Mock + pcDNA, φ*p* < 0.01 compared with miR-509-3p + pcDNA. **E** Colony formation assay on cell growth of A549-derived stem cells after 15 days culture, *p < 0.01 compared with Mock + pcDNA, φ*p* < 0.01 compared with miR-509-3p + pcDNA. **F** qRT-PCR analysis of growth-associated f genes (Cyclin A1, Cyclin B1, and PCNA) in stem cells of A549 cells, *p < 0.01 compared with Mock + pcDNA, φ*p* < 0.01 compared with miR-509-3p + pcDNA

To determine whether YAP1 is required for the function of miR-509-3p, we overexpressed YAP1 and miR-509-3p in A549-dereived stem cells. It was found that overexpression of YAP1 reversed the inhibitory effect of miR-509-3p on stemness evidenced by sphere formation and restoring expression of the stemness-associated genes SOX2, CD44, and CD133 in A549-dereived stem cells (Fig. 7A, B). Overexpression of YAP1significantly attenuated miR-509-3p-induced apoptosis by restoring miR-509-3p-controlled expression of apoptotic genes Bax, Bcl-2, caspase-3 (Fig. 7C, D). Furthermore, YAP1 overexpression abolished the suppressive effect of miR-509-3p on cell growth in A549-dereived stem cells via recovering expression of cell growth-related genes Cyclin A1, Cyclin B1, and PCNA (Fig. 7E, F).

## Discussion

Increasing evidence have demonstrated that cancer stem cells participate in the aggressive and destructive behaviors of lung cancers including NSCLC, a main cause of cancer-induced death worldwide [2, 3, 35, 36]. The cancer stem cells significantly increase the recurrence of NSCLC after surgery or radiotherapy [4–6]. The cancer stem cells possess the properties of self-renewal, cancer initiation, and cancer progression [7]. Cancer stem cells also aggravate multi-drug resistance in lung cancer [8]. Therefore, exploring cancer stem cell-related potential molecular mechanism in NSCLC progression is critical, as it would contribute to the treatment of patients with NSCLC.

It has been reported that a variety of lncRNAs are involved in the progression of NSCLC [14, 15, 37, 38]. As reported in previous studies, ASAP1-IT1 is upregulated in NSCLC and promotes cancer proliferation, invasion and migration [22]. Consistent with this report, our preliminary sequencing data shows that ASAP1-IT1 was one of the top 20 up-regulated lncRNA in NSCLC with poor clinical outcome (data not shown). Moreover, ASAP1-IT1 was validated to be upregulated in NSCLC tumors, cancer cells, and A549 cell spheres. Cancer stem cells was reported to efficiently affect multi-drug resistance in NSCLC [8, 39–42]. Here we demonstrated that knockdown of ASAP1-IT1 significantly suppressed cancer cell stemness of NSCLC cells and increase chemoresistance to cisplatin in NSCLC cells. ASAP1-IT1 was also overexpressed in A549/R cells, suggesting that ASAP1-IT1 could be associated with cisplatin resistance. Knockdown of ASAP1-IT1significantly inhibited cancer stem cell colony formation and A549 cancer cell stemness. Further studies demonstrated that knockdown of ASAP1-IT1 suppressed expression of stem cell biomarkers SOX2, CD34, and CD133 in A549-dereived stem cells. Then, downregulating ASAP1-IT1 expression decreased expression of cell growth-associated genes Cyclin A1, Cyclin B1, and PCNA, and elevated expression of cell apoptosis related genes Bax and caspase-3 and reduced anti-apoptotic Bcl-2 expression in A549-dereived stem cells. The in vivo experiments proved that knockdown of ASAP1-IT1 significantly inhibited tumor growth in nude mice. Thus, these in vitro and in vivo findings suggested that ASAP1-IT1 acted as an oncogene to regulate cancer cell stemness, cell growth and cell apoptosis in NSCLC. Down-regulation of ASAP1-IT1 could be a good strategy to fight against NSCLC.

LncRNAs exert their activity in cancers often by functioning as sponges of miRNAs [15, 43]. In this study, ASAP1-IT1 was firstly predicted to interact with miR-509-3p. The interaction between ASAP1-IT1 and miR-509-3p was proved by luciferase RIP assays. Previous studies have revealed that miR-509-3p inhibits tumor growth in multiple types of cancers. For example, miR-509-3p improves sensitivity of cancer cells to platinum in ovarian cancer [23, 24, 44], and it depresses cell proliferation and invasion via down-regulating X-linked inhibitor of apoptosis in glioma [28]. MiR-509 promotes hepatoma progression by activating NF-κB (nuclear factor kappa B) signaling pathway. However, the role of miR-509-3p in the stemness of NSCLC has not been investigated. In consistent with previous studies, miR-509-3p was significantly down-regulated in NSCLC tissues, NSCLC cells, A549 spheres, and in A549/R cells. Overexpression of miR-509-3p suppressed sphere formation and cell growth, and increased cell apoptosis of A549-dereived stem cells. Also, overexpression of miR-509-3p decreased expression of SOX2, CD44, and CD133, and enhanced expression of Bax and caspase-3 while repressed Bcl-2 expression in cancer cells. Furthermore, overexpression of miR-509-3p suppressed tumor growth in nude mice. These results implied that miR-509-3p was a tumor suppressor, and overexpression of miR-509-3p could offer a novel approach to prevent NSCLC progression. Furthermore, overexpression of ASAP1-IT1 significantly blocked overexpression of miR-224-3p-mediated inhibition of cancer stem cell-like properties and cell growth of A549-dereived stem cells both in vitro and in vivo. Additionally, overexpression of ASAP1-IT1 abolished miR-509-3p-induced cancer cell apoptosis of A549-dereived stem cells both in vitro and in vivo, indicating that the interaction between ASAP1-IT1 and miR-509-3pis involved in regulation of cancer cell stemness and NSCLC progression.

Bioinformatics analysis showed that miR-509-3p could target the 3′UTR of YAP1, which is an oncogene in the Hippo signaling pathway involved in human cancers [34]. Pan et al*.* reported that miR-509-3p attenuates ovarian cancer cellular migration and formation of multi-cellular spheroids by targeting YAP144. Our study also demonstrates that miR-509-3p abrogates cancer cell stemness via downregulation of YAP1. Our results were in consistent with the previous studies which supported that YAP1 is an oncogene and aggravates drug resistance and cancer cell stemness in NSCLC [29–33, 45, 46]. Moreover, we also observed that interaction of ASAP1-IT1 with miR-509-3p led to reciprocally inhibition. These findings demonstrated that YAP1 is involved in ASAP1-IT1/miR-509-3p interaction and plays an important role in cancer cell stemness and progression of NSCLC.

## Conclusion

In conclusion, ASAP1-IT1 is up-regulated in NSCLC and promotes cancer cell stemness by suppressing miR-509-3p. YAP1 is modulated by ASAP1-IT1 and miR-509-3p in A549-dereived stem cells. ASAP1-IT1 and miR-509-3p could be potential therapeutic targets of NSCLC (Additional file 1: Figure S1).

## Supplementary Information

**Additional file 1.** ASAP1-IT1 increases cancer cell stemness by regulating miR-509-3p/YAP1 signal pathway in NSCLC cells.

## Data Availability

The datasets supporting the conclusions of this article are included within the article and could be obtained from the corresponding author if necessary.

## Abbreviations

ASAP1-IT1: ASAP1 Intronic Transcript 1
NSCLC: Non-small cell lung cancer
CSCs: Cancer stem cells
lncRNAs: Long noncoding RNAs
miR-509-3p: MicroRNA-509-3p
RIP: RNA immunoprecitation
H&E: Hematoxylin–eosin
CCK-8: Cell counting kit‐8
Linc00673/00473: Long intergenic non-protein coding RNA 673/00473
cAMP/CREB: Adenosine monophosphate/cAMP-response element binding protein
LKB1: Liver kinase B1
DGC5: DiGeorge syndrome critical region gene 5
FENDFRR: FOXF1 adjacent non-coding developmental regulatory RNA
MDR1: Multi-drug resistance gene 1
PTEN/AKT: Phosphatase and tensin homolog/AKT serine/threonine kinase 1
XIAP: X-linked inhibitor of apoptosis
YAP1: Yes associated protein 1
