# Supplementary figures and images for: LncRNA ASAP1-IT1 enhances cancer cell stemness via regulating miR-509-3p/YAP1 axis in NSCLC
Source: Cancer Cell Int. 2021 Oct 29;21:572. doi: 10.1186/s12935-021-02270-7 (PMC8555224; doi:10.1186/s12935-021-02270-7)

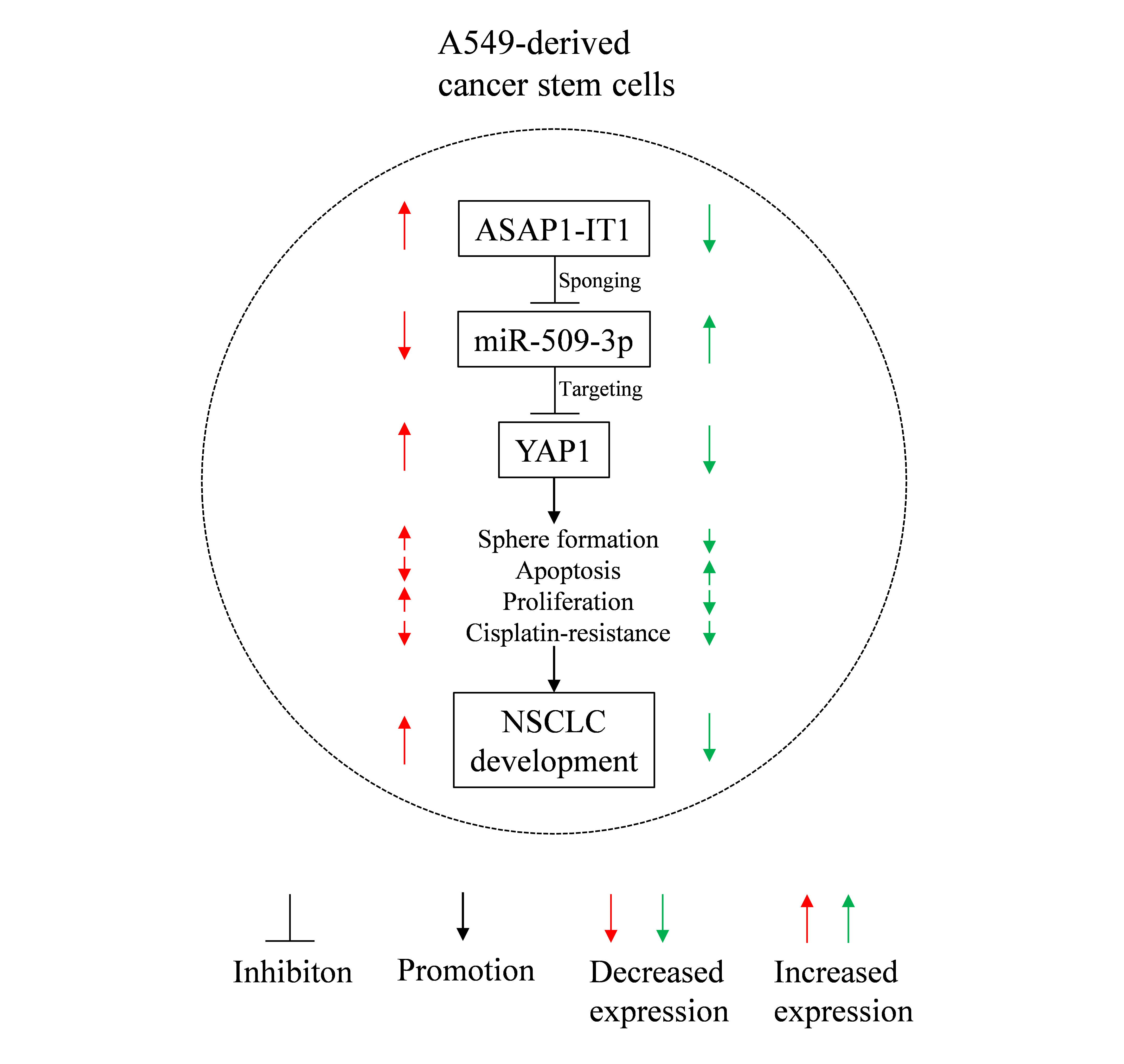

Supplement: Supplementary file 1 — Additional file 1. ASAP1-IT1 increases cancer cell stemness by regulating miR-509-3p/YAP1 signal pathway in NSCLC cells. [file 12935_2021_2270_MOESM1_ESM.tif]
